# Efficacy and safety of Jintiange in the treatment of osteoporosis: a systematic review and meta-analysis

**DOI:** 10.3389/fphar.2025.1592184

**Published:** 2025-07-14

**Authors:** Yicong Man, Junfu Na, Hongxu Wang, Fang Lan, Liying Yu

**Affiliations:** ^1^ Department of Orthopedics and Traumatology, Lishui Hospital of Traditional Chinese Medicine, Lishui, Zhejiang, China; ^2^ Department of Orthopedics and Traumatology, Affiliated Hospital of Liaoning University of Traditional Chinese Medicine, Shenyang, Liaoning, China; ^3^ Department of Acupuncture and Moxibustion, Lishui Hospital of Traditional Chinese Medicine, Lishui, Zhejiang, China

**Keywords:** Jintiange, artificial tiger bone powder, osteoporosis, BMD, safety

## Abstract

**Objective:**

To evaluate the efficacy and safety of Jintiange in osteoporosis treatment via systematic review and meta-analysis, thereby presenting more supporting evidence.

**Methods:**

Up to 27 October 2024, PubMed, Web of Science, Cochrane Library, Embase, China National Knowledge Infrastructure (CNKI), and Wanfang were searched for studies on the use of Jintiange/artificial tiger bone powder in osteoporosis treatment. Studies were selected based on predefined eligibility criteria. Outcome measures encompassed bone mineral density (BMD), pain scores, adverse event (AE), fracture incidence, serum calcium, and phosphorus concentrations, as well as bone resorption and formation markers.

**Results:**

This study included 18 studies involving 21 trials on 2,580 patients (1,303 in the Jintiange group and 1,277 in the control group). A pooled analysis demonstrated that, in comparison to the control group, the Jintenge group achieved significantly greater improvements in BMD at various anatomical sites, including the lumbar spine (SMD = 0.52), femoral neck (SMD = 0.31), greater trochanter (SMD = 0.59), and Ward’s triangle (SMD = 0.94). In addition, the Jintenge group exhibited a greater reduction in Visual Analogue Scale (VAS) scores (SMD = −0.87). No significant differences were observed between the two groups in terms of AEs or incidence of fractures. The level of bone Gla protein (BGP) was significantly higher in the Jintenge group compared with the control group (SMD = 1.28), whereas there were insignificant intergroup differences in serum calcium and phosphorus concentrations or changes in the type I collagen carboxy-terminal peptide (CTX). Sensitivity analysis revealed inconsistent results of Procollagen type I N-terminal propeptide (PINP) and bone alkaline phosphatase (BALP).

**Conclusion:**

Jintiange possibly improves BMD and alleviates pain in osteoporosis patients, with a favorable safety profile. Prolonged treatment duration (exceeding 6 months) yields greater therapeutic benefit than shorter courses, and the combination of Jintiange with standard therapies demonstrates superior efficacy relative to Jintiange alone. However, in consideration of the limitations of the present study, further high-quality investigations are necessitated to strengthen the evidence base and to elucidate the long-term efficacy, safety, and impact of this agent on fracture incidence.

**Systematic Review Registration:**

https://www.crd.york.ac.uk/PROSPERO/, identifier CRD42025630527.

## 1 Introduction

Osteoporosis, the most prevalent bone metabolic disorder worldwide, primarily features the decrease in bone mass and destruction of bone microstructure, thereby giving rise to elevated risks of bone fragility and fractures (LeBoff et al., 2022a). Old people and postmenopausal women are particularly susceptible to this condition, and with the increasing population and aging demographics, the global economic burden will continue to rise (Barcelos et al., 2023). According to the epidemiological survey conducted in America from 2017 to 2018, the incidence of osteoporosis among the population aged 50 and more has risen from 9.4% a decade ago to 12.6%. Approximately 2 million fragility fractures are reported annually, resulting in an expenditure exceeding USD 17 billion (Sarafrazi et al., 2021). Pharmacological treatment is its primary therapeutic approach. Mainstream medications include calcium supplements, vitamin D, bisphosphonates, denosumab, and teriparatide, among others. However, adverse events (AEs) and the potential long-term hazards of these treatments often reduce patient adherence and impact treatment outcomes (Ensrud and Crandall, 2024). Therefore, the continuous optimization of pharmacological prevention and treatment strategies remains crucial in this field globally.

Jintiange is a commercial Chinese polyherbal preparation whose active ingredient is artificial tiger bone powder. In ancient China, tiger bone, sourced from the skeleton of *Panthera tigris* L., a member of the Felidae family, was regarded as a precious traditional Chinese medicinal material. According to traditional Chinese medicine (TCM) theory, tiger bone possesses properties that dispel wind, alleviate pain, strengthen bones, and reinforce tendons. However, in modern times, with the advancement of wildlife conservation legislation, the use of tiger bone in medicine was officially prohibited in 1993. To balance the imperative of wildlife protection with clinical demand, Chinese researchers conducted chemical analyses of natural tiger bone to identify viable artificial alternatives. Against this backdrop, Jintiange (artificial tiger bone powder) was successfully developed in 2003 (Xing et al., 2013). In order to replicate both the inorganic and organic components of natural tiger bone, the formulation of Jintiange utilizes legally and sustainably sourced bones from domesticated food animals, including *Sus scrofa* domestica L., *Capra hircus* Linnaeus, and Cervus nippon Temminck. Its composition includes approximately 18% calcium, 8% phosphorus, as well as peptides and proteins (Liu et al., 2023). Studies have demonstrated that the physicochemical and biochemical properties of Jintiange, including nitrogen content, dynamic viscosity, and optical rotation, closely approximate those of natural tiger bone. Moreover, no significant differences have been observed in terms of pharmacological activity. Jintiange exhibits anti-inflammatory, analgesic, bone-healing, and bone metabolism-improving effects (Guo et al., 2006). Therefore, it was approved as a Class I new drug by the China Food and Drug Administration (CFDA; China, Z20030080) and has since been widely applied in the treatment of osteoporosis, osteoarthritis, among others (Clinical Guidelines for the Treatment of Osteoporosis with Proprietary Chinese Medicines, 2021). A clinical randomized controlled trial (RCT) by Shu and Zhang (2022) indicated that Jintiange could increase bone mineral density (BMD), alleviate pain, and increase functional scores. Furthermore, Liang et al. (2022a) conducted a 52-week treatment study involving 400 osteoporosis patients recruited between 2016 and 2019 and proved that Jintiange ameliorated BMD, muscle strength, and lower limb balance, contributing to a lowered risk of falls.

Despite plenty of clinical RCTs supporting the efficacy of Jintiange in osteoporosis treatment, no international evidence-based meta-analysis has yet been carried out to synthesize the data. Moreover, the quality of the studies varies, and their conclusions are not entirely consistent. Therefore, this study seeks to aggregate data from eligible RCTs via a systematic review and meta-analysis and present higher-quality, more comprehensive evidence for the efficacy and safety of Jintiange in osteoporosis treatment.

## 2 Materials and methods

### 2.1 Registration and protocol

Before commencement, our study was registered on the PROSPERO website (Registration No.: CRD42025630527). This systematic review strictly followed the Preferred Reporting Items for Systematic Reviews and Meta-Analysis (PRISMA) guidelines (Page et al., 2021) to ensure the reliability and reproducibility of the research.

### 2.2 Search strategy

Four English databases (PubMed, Web of Science, Cochrane Library, and Embase) and two Chinese databases (CNKI and Wanfang) were thoroughly retrieved for articles related to the utilization of Jintiange/artificial tiger bone powder in osteoporosis treatment, from their inception to 27 October 2024. The search terms included “Jintiange OR Tiger Bone Powder” and “Osteoporosis”. No restrictions were imposed on language or region. The results were managed via EndNote 21. In addition, references of articles meeting the eligibility criteria were manually checked to ensure completeness. The search strategy is detailed in Supplementary Appendix 1.

### 2.3 Screening strategy

After de-duplication, articles were checked as per the eligibility criteria outlined below, based on titles, keywords, abstracts, and complete textual content.

The inclusion criteria were: (1) Participants were patients with osteoporosis or low bone mass (including postmenopausal, old people, chronic kidney disease, glucocorticoid use, hypertension, inflammatory bowel disease, diabetes, osteoarthritis, and related conditions); (2) The intervention in the Jintiange group was either Jintiange (Jintiange Capsules, composed of artificial tiger bone powder and manufactured by Ginwa Enterprise (Group) Inc., Xi’an, China) used alone or combined with conventional biomedicine, while the control group received conventional biomedicine or placebo; (3) At least one of the following outcome measures was reported: BMD, pain score, number of AEs, bone metabolism markers, among others; (4) The study design was a clinical RCT; (5) To ensure the quality of eligible literature, Chinese studies must be publications in core journals (as defined by Peking University’s Chinese Core Journal Overview or Chinese Scientific and Technological Core Journals).

The following studies were ostracized: (1) Reviews, animal or cell experiments, conference abstracts, or articles without full texts; (2) Articles without clear diagnosis criteria for osteoporosis; (3) Studies on osteoporotic fractures; (4) Articles with interventions involving other traditional Chinese medicines (TCM), botanical drug, or therapies; (5) Those with unavailable data that could not be acquired from the original authors.

The most comprehensive article was selected in case of duplicate publications, and studies originating from the same research project were merged based on their content.

### 2.4 Data extraction and handling

Two researchers independently executed article screening and data extraction. Dissents were settled by a third researcher. Data were entered into Microsoft Excel and encompassed the first author, publication year, region, design, population characteristics, sample size, average age, sex ratio, intervention approach, intervention duration, and outcome measures (BMD, pain score, number of AEs, and bone metabolism markers). For continuous outcome variables, the changes in values before and after treatment were calculated and recorded as mean ± standard deviation (Luo et al., 2018). For studies in which data could not be obtained, attempts were made to contact the corresponding authors to acquire the original data.

### 2.5 Quality assessment

Two researchers independently examined the quality of eligible studies utilizing the evidence quality assessment tool in RevMan5.4 and leveraged the risk of bias (ROB) tool for RCTs. This tool includes random sequence generation, allocation concealment, participant and personnel blinding, blinding of outcome assessment, incomplete outcome data, reporting bias, and other biases (presence of conflicts of interest, baseline imbalance, and regional or environmental bias). Each was evaluated and rated as low, high, or unclear risk based on the content of the encompassed studies.

### 2.6 Data analysis method

Data were synthesized and analyzed via RevMan5.4 and Stata15. For continuous variables, standardized mean difference (SMD) was employed as the effect size, while risk ratio (RR) was utilized for categorical variables, both with 95% confidence intervals (CIs). Heterogeneity was detected via the Cochran Q test and I^2^ statistic (Bowden et al., 2011; Wang et al., 2024). Significant heterogeneity was defined as I^2^ > 50% or P < 0.05. A random-effects model was leveraged to calculate the pooled effect size. Forest plots visually presented the results. Sensitivity analyses and subgroup analyses were conducted to assess the robustness of the results and to identify potential sources of heterogeneity. Publication bias was evaluated qualitatively and quantitatively via funnel plots and Egger’s test (Egger et al., 1997). Furthermore, in accordance with the Grading of Recommendations Assessment, Development, and Evaluation (GRADE) approach, the quality of evidence for each outcome was assessed and categorized as high moderate, low, or very low to facilitate the formulation of conclusions (Guyatt et al., 2011).

## 3 Results

### 3.1 Literature selection process

**FIGURE 1:**
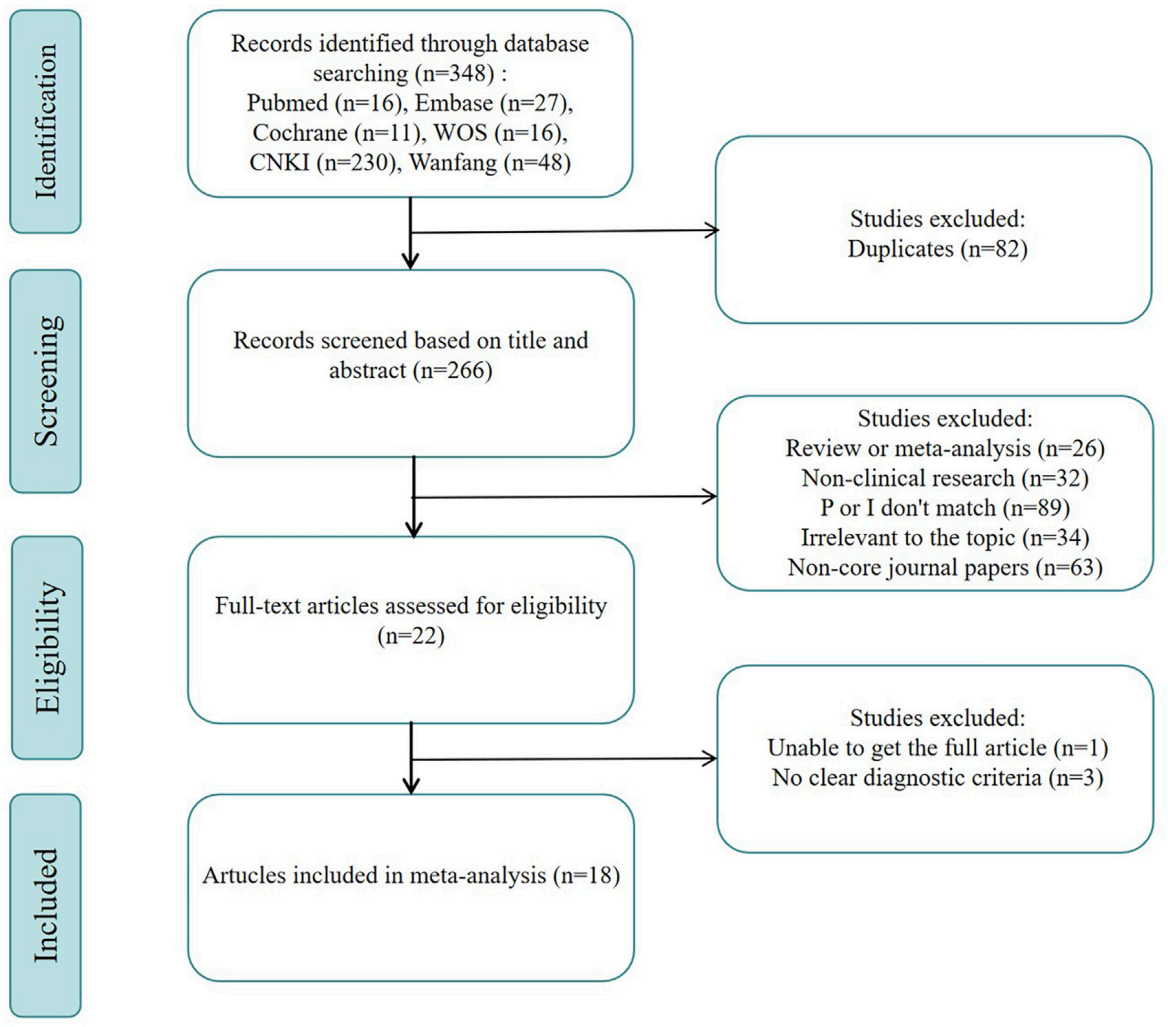
Flowchart of the systematic search and selection process.

A systematic search across six databases initially yielded 348 relevant articles. After 82 duplicates were removed, 266 articles remained and were screened by titles and abstracts. As per the eligibility criteria, 244 articles were ostracized, including 26 review or meta-analysis articles, 32 non-clinical studies (such as animal experiments, cell trials, and pharmacological research), 89 studies with non-eligible subjects or interventions (for example, osteoporosis with fractures or interventions involving other TCM or treatments), and 34 studies unrelated to the research topic (including notifications, announcements, and conference papers). 63 articles published in non-core journals were eliminated after a search through China National Knowledge Infrastructure (CNKI) (www.cnki.net). Subsequently, full-text retrieval and detailed review were conducted for the rest 22 articles. Of these, one article could not be retrieved in full text, and three studies were excluded due to unclear osteoporosis diagnoses among participants. Ultimately, 18 articles were encompassed for final analysis (Zhang et al., 2014; Du and Shao, 2015; Fan et al., 2015; Gan et al., 2015; He et al., 2015; Xu et al., 2015; Zeng et al., 2016; Pan et al., 2016; Zhu and Song, 2016; Cai et al., 2017; Fu et al., 2017; Huang et al., 2017; Wei and Zhang, 2017; Dai et al., 2018; Wang et al., 2022; Liang et al., 2022b; Chen et al., 2023; Gu et al., 2024). The selection process is presented in Figure 1.

### 3.2 Study characteristics

Among the 18 encompassed studies, two articles by Wang et al. (2022) and Liang et al. (2022b) originated from the same large multi-center RCT but reported different outcome measures. These were merged for content. Therefore, 17 clinical studies examining the utilization of Jintiange for osteoporosis treatment were included for further analysis. All of them were RCTs carried out in China from 2009 to 2024. Regarding patient characteristics, seven studies included postmenopausal women with osteoporosis, of which two also involved patients with diabetes. Two studies focused on old patients with osteoporosis, with one study involving patients with hypertension. The remaining eight studies did not classify osteoporosis but encompassed one study with early-stage diabetic nephropathy and two studies with knee osteoarthritis. No studies on secondary osteoporosis were encompassed. Among the 17 studies, two trials by Du Jianru involved two experimental groups (Jintiange monotherapy group and Jintiange combined with biomedicine group) and one control group, while Gan Qiang set up three experimental groups (Jintiange monotherapy group and two Jintiange combined with biomedicine groups) and one control group. These studies were analyzed separately for each experimental group and control group as distinct studies. In addition, He Baoyu et al. split the patients into two age groups when comparing BMD data. Therefore, the encompassed participants were divided into two groups for analysis in this study. In total, 21 studies were eligible for our analysis and involved 2,580 patients, with 1,303 in the experimental cohort and 1,277 in the control cohort. The average age was 55.6–71. The interventions included Jintiange, calcium supplements, calcitriol, calcitonin, and bisphosphonates, with treatment periods ranging from 1 to 12 months. The study characteristics are detailed in Table 1.

**TABLE 1:** Characteristics of studies included for meta-analyses.

Author	Region	Population	Intervention	Jintiange composition	Control	Treatment duration (M)	Patients(n)	Controls(n)	Female (%)	Mean age
Zhang et al. (2014)	China	Elderly osteoporosis	Jintiange capsule + salmon calcitonin injection	Artificial tiger bone powder	Salmon calcitonin injection	6	23	23	32.6	63.5
Du and Shao (2015)	China	DN with osteoporosis	Jintiange capsule	Artificial tiger bone powder	Caltrate with vitamin D tablets + calcitriol soft capsules + alendronate sodium enteric coatel tablets	5.5	34	34	NA	NA
Du and Shao (2015)	China	DN with osteoporosis	Jintiange capsule + caltrate with vitamin D tablets + calcitriol soft capsules + alendronate sodium enteric coatel tablets	Artificial tiger bone powder	Caltrate with vitamin D tablets + calcitriol soft capsules + alendronate sodium enteric coatel tablets	5.5	34	34	NA	NA
Fan et al. (2015)	China	Primary osteoporosis	Jintiange capsule + caltrate with vitamin D tablets	Artificial tiger bone powder	Caltrate with vitamin D tablets	3	50	50	79	62.7
Gan et al. (2015)	China	Postmenopausal osteoporosis	Jintiange capsule	Artificial tiger bone powder	Caltrate with vitamin D tablets + alendronate sodium tablets	6	59	58	100	57.8
Gan et al. (2015)	China	Postmenopausal osteoporosis	Jintiange capsule + caltrate with vitamin D tablets	Artificial tiger bone powder	Caltrate with vitamin D tablets + alendronate sodium tablets	6	58	58	100	57.8
Gan et al. (2015)	China	Postmenopausal osteoporosis	Jintiange capsule + caltrate with vitamin D tablets + alendronate sodium tablets	Artificial tiger bone powder	Caltrate with vitamin D tablets + alendronate sodium tablets	6	63	58	100	57.8
He et al. (2015)	China	Primary osteoporosis	Jintiange capsule	Artificial tiger bone powder	caltrate with vitamin D tablets	9	46	44	57.5	64.6
He et al. (2015)	China	Primary osteoporosis	Jintiange capsule	Artificial tiger bone powder	Caltrate with vitamin D tablets	9	34	36	57.5	64.6
Xu et al. (2015)	China	Primary osteoporosis	Jintiange capsule + caltrate with vitamin D tablets + calcitriol soft capsules + zoledronic acid injection	Artificial tiger bone powder	Caltrate with vitamin D tablets + calcitriol soft capsules	12	23	21	NA	56.7
Zeng et al. (2016)	China	Postmenopausal osteoporosis	Jintiange capsule + calcium carbonate tablets + alfacalcidol capsules	Artificial tiger bone powder	calcium carbonate tablets + alfacalcidol capsules + alendronate sodium capsule	6	52	34	100	59.3
Pan et al. (2016)	China	DN with postmenopausal osteoporosis	Jintiange capsule + caltrate with vitamin D tablets + zoledronic acid injection	Artificial tiger bone powder	Caltrate with vitamin D tablets + zoledronic acid injection	6	70	70	100	67.2
Zhu and Song (2016)	China	Knee osteoarthritis with Osteoporosis	Jintiange capsule + elcatonin injection	Artificial tiger bone powder	Aceclofenac tablets + elcatonin injection	1	45	45	72.2	68.6
Cai et al. (2017)	China	Elderly hypertension with osteoporosis	Jintiange capsule + caltrate with vitamin D tablets	Artificial tiger bone powder	Caltrate with vitamin D tablets	12	72	72	38.2	71
Fu et al. (2017)	China	Postmenopausal osteoporosis	Jintiange capsule + caltrate with vitamin D tablets + calcitriol soft capsules + zoledronic acid injection	Artificial tiger bone powder	Caltrate with vitamin D tablets + calcitriol soft capsules + zoledronic acid injection	12	33	33	100	67.1
Huang et al. (2017)	China	Postmenopausal osteoporosis	Jintiange capsule + caltrate with vitamin D tablets	Artificial tiger bone powder	Caltrate with vitamin D tablets	2.8	86	86	100	56.8
Wei and Zhang (2017)	China	Primary osteoporosis	Jintiange capsule + calcium carbonate tablets + vitamin D	Artificial tiger bone powder	calcium carbonate tablets + vitamin D	6	38	38	53.9	69.8
Dai et al. (2018)	China	DM with postmenopausal osteoporosis	Jintiange capsule + caltrate with vitamin D tablets + zoledronic acid injection	Artificial tiger bone powder	Caltrate with vitamin D tablets + zoledronic acid injection	6	80	80	100	67.8
Wang et al. (2022)	China	Primary osteoporosis	Jintiange capsule + calcium carbonate tablet mimetic agent + alfacalcidol capsules	Artificial tiger bone powder	Jintiange capsule mimetic agent + calcium carbonate tablets + alfacalcidol capsules	12	199	200	86.7	63.1
Chen et al. (2023)	China	Knee osteoarthritis with Osteoporosis	Jintiange capsule	Artificial tiger bone powder	Jintiange capsule mimetic agent	11.1	124	123	77.7	55.6
Gu et al. (2024)	China	Postmenopausal osteoporosis	Jintiange capsule + caltrate with vitamin D tablets + alfacalcidol capsules + alendronate sodium tablets	Artificial tiger bone powder	Caltrate with vitamin D tablets + alfacalcidol capsules + alendronate sodium tablets	12	80	80	100	58.4

### 3.3 ROB assessment results

**FIGURE 2:**
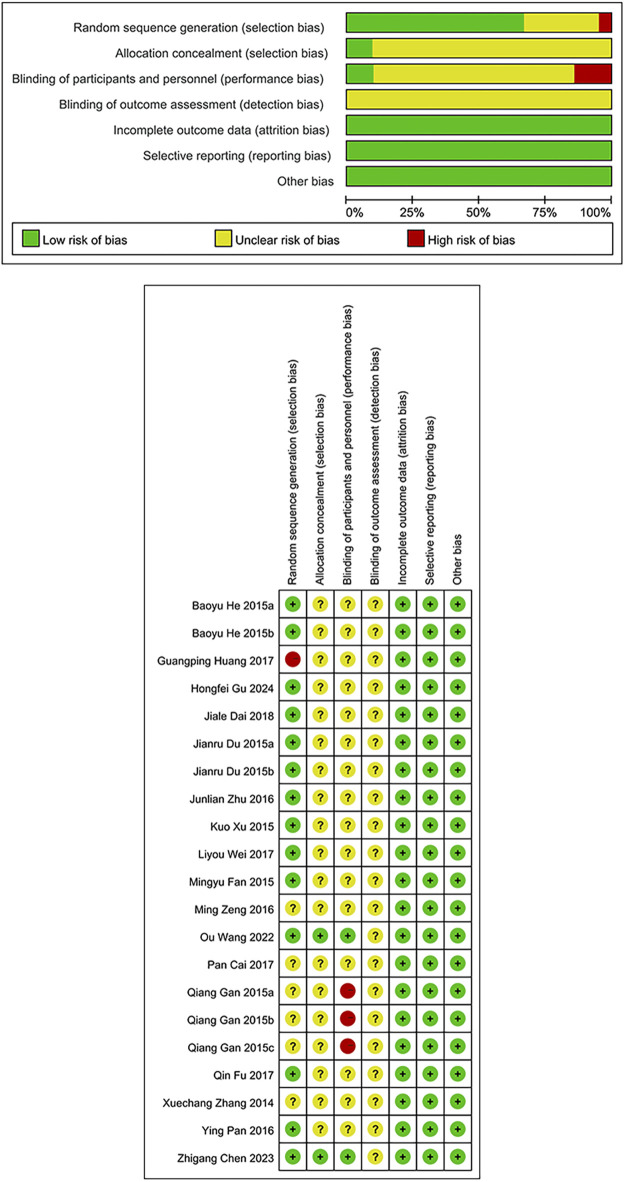
Risk of bias graph/Risk of bias summary.

Among the 21 studies included in the analysis, 14 studies (Du and Shao, 2015; Fan et al., 2015; He et al., 2015; Xu et al., 2015; Pan et al., 2016; Zhu and Song, 2016; Fu et al., 2017; Wei and Zhang, 2017; Dai et al., 2018; Wang et al., 2022; Chen et al., 2023; Gu et al., 2024) utilized appropriate randomization methods (random number table, random envelope, random block, and coin toss methods), which were rated as being of low risk. One study (Huang et al., 2017) that grouped participants based on visit time was regarded as possessing high risk, and the rest that did not specify randomization methods were deemed as having unclear risk. The primary methodological limitations lie in the processes of allocation concealment and blinding. Only two studies (Wang et al., 2022; Chen et al., 2023 )implemented allocation concealment and were consequently assessed as having a low risk of bias; the remaining studies did not report on allocation concealment and were therefore judged to have an unclear risk. Similarly, only these two studies employed double-blinding and were assessed as low risk in this domain. In contrast, three studies (Gan et al., 2015) explicitly informed patients of their treatment allocation, leading to a high risk of bias. The remaining studies did not mention the use of blinding and were thus evaluated as having an unclear risk. None of the included studies reported blinding in outcome assessment, resulting in an unclear risk of bias in this domain across all studies. In consideration of outcome completeness, selective reporting, and other potential biases, no major concerns were identified. Therefore, there was a low risk of bias. The ROB assessment is illustrated in Figure 2.

### 3.4 BMD changes

BMD serves as a crucial indicator for assessing fracture risk at corresponding skeletal sites. In light of this association, the present study conducted a meta-analysis of BMD changes stratified by different anatomical locations. Included studies reported BMD changes at the lumbar spine, femoral neck, greater trochanter, Ward’s triangle, total hip, distal radius, and forearm. However, only a single study measured BMD changes at the distal radius and forearm, failing to meet the minimum study number required for meta-analytic synthesis; therefore, these sites were excluded from the quantitative synthesis. The pooled results for the remaining sites are presented as follows.

#### 3.4.1 Lumbar spine

**FIGURE 3:**
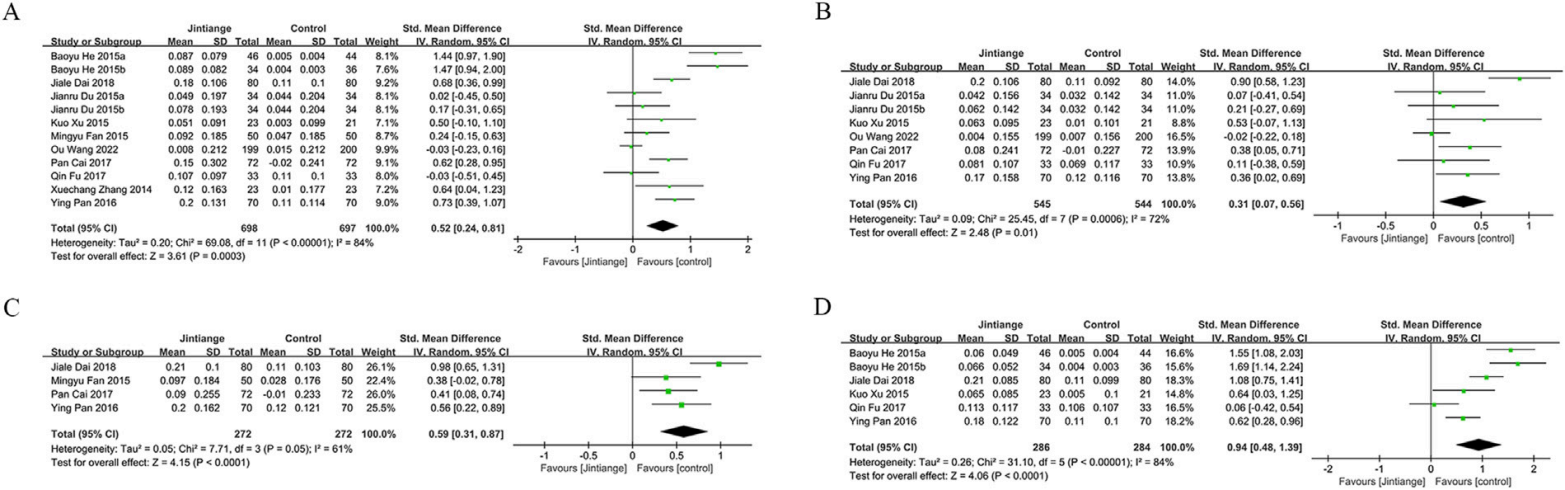
Forest plots illustrate the impact of Jintiange vs the control group on **(A)** lumbar spine BMD, **(B)** femoral neck BMD, **(C)** greater trochanter BMD, and **(D)** Ward’s triangle BMD.

Data on lumbar spine BMD changes were derived from 12 studies, including 1,395 patients (698 in the Jintiange group and 697 in the control group). The pooled analysis showed that Jintiange significantly enhanced lumbar spine BMD, with a more pronounced effect in comparison to the control group (SMD: 0.52; 95% CI: 0.24, 0.81; p = 0.0003). The heterogeneity was significant (I^2^ = 84%, p < 0.00001) (Figure 3A). The funnel plot and Egger’s test did not indicate publication bias (p = 0.106) (Figure 6A).

#### 3.4.2 Femoral neck

Eight studies examined changes in femoral neck BMD, encompassing 1,089 patients (545 in the Jintiange cohort and 544 in the control cohort). The pooled analysis demonstrated that Jintiange resulted in a more significant enhancement of BMD in the femoral neck when contrasted with the control cohort (SMD: 0.31; 95% CI: 0.07, 0.56; p = 0.01), with significant heterogeneity (I^2^ = 72%, p = 0.0006) (Figure 3B). There was no publication bias in the funnel plot and Egger’s test (p = 0.388) (Figure 6B).

#### 3.4.3 Greater trochanter

Four studies reported data on BMD changes at the greater trochanter, involving 544 patients (272 in each group). The pooled analysis indicated that the Jintiange group displayed a markedly greater rise in BMD at the greater trochanter than the control cohort (SMD: 0.59; 95% CI: 0.31, 0.87; p < 0.0001), with substantial heterogeneity (I^2^ = 61%, p = 0.05) (Figure 3C). The funnel plot indicated slight publication bias (Figure 6C), though Egger’s test did not reveal statistically significant publication bias (p = 0.515).

#### 3.4.4 Ward’s triangle

Data on changes in BMD at Ward’s triangle were from six studies involving 570 patients (286 in the Jintiange cohort and 284 in the control cohort). The pooled analysis showed that Jintiange notably increased BMD at Ward’s triangle, with a more pronounced increase than the control group (SMD: 0.94; 95% CI: 0.48, 1.39; p < 0.0001), exhibiting significant heterogeneity (I^2^ = 84%, p < 0.00001) (Figure 3D). Funnel plot and Egger’s test suggested no publication bias (p = 0.822) (Figure 6D).

### 3.5 Visual Analogue Scale (VAS) score changes

**FIGURE 4:**
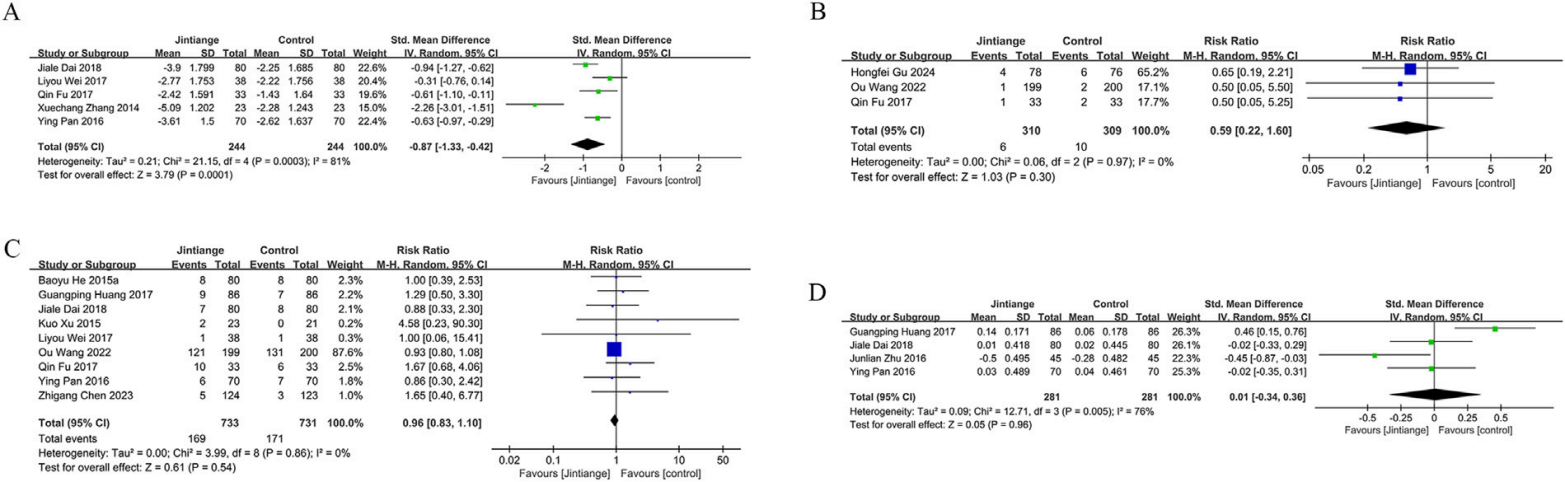
Forest plots illustrate the impact of Jintiange vs the control group on **(A)** VAS score changes, **(B)** fracture incidence, **(C)** AE incidence, and **(D)** serum calcium.

Five studies measured VAS scores before and after treatment in 488 patients (244 in each group). The pooled analysis showed a decrease in VAS scores for both groups, with a more significant reduction in pain among Jintiange receivers (SMD: 0.87; 95% CI: 1.33, −0.42; p = 0.0001), showing considerable heterogeneity (I^2^ = 81%, p = 0.0003) (Figure 4A). The funnel plot suggested slight publication bias (Figure 6E), although Egger’s test showed no statistically significant publication bias (p = 0.385).

### 3.6 Incidence of fractures

Fragility fractures represent the most severe complication of osteoporosis. Only three studies reported the incidence of fragility fractures, with no fractures observed during the study period in the other studies. These studies involved 619 patients (310 in the Jintiange cohort and 309 in the control cohort). The pooled analysis indicated similar fracture incidences between groups (RR: 0.59; 95% CI: 0.22, 1.60; p = 0.3), with no notable heterogeneity (I^2^ = 0%, p = 0.97) (Figure 4B). Statistical (Egger’s test, p = 0.022) and visual (Figure 6F) evidence indicated publication bias.

### 3.7 Incidence of AEs

Nine studies reporting on AEs were included and involved 1,464 patients (733 in the Jintiange group and 731 in the control group). AEs occurring at least twice or more were defined as high-frequency AEs. In the Jintiange group, the high-frequency AEs included dry mouth, constipation, fever, dizziness, nausea and vomiting, abdominal pain, and myalgia, whereas in the control group, high-frequency AEs comprised dry mouth, constipation, fever, nausea and vomiting, and myalgia. A report on AEs is detailed in Supplementary Appendix 2. The results showed an insignificant difference in the incidence of AEs across the Jintiange and control cohorts (RR: 0.96; 95% CI: 0.83, 1.10; p = 0.54), with low heterogeneity (I^2^ = 0%, p = 0.86) (Figure 4C). The funnel plot revealed slight publication bias (Figure 6G), but statistically significant publication bias was not detected in Egger’s test (p = 0.056).

### 3.8 Changes in bone metabolism markers

Bone metabolism markers refer to hormones regulating calcium and phosphorus metabolism, serum calcium, phosphorus, magnesium, sex hormones, bone resorption markers, and bone formation markers, among others (Kellar et al., 2023; Schini et al., 2023). The following markers were synthesized and analyzed in the encompassed studies.

#### 3.8.1 Serum calcium and phosphorus

Four studies reported serum calcium data on 562 patients (281 in the Jintiange group and 281 in the control group). The difference was insignificant across groups (SMD: 0.23; 95% CI: 0.34, 0.36; p = 0.96), although statistically significant heterogeneity was noted (I^2^ = 76%, p = 0.005) (Figure 4D). Neither Egger’s test (p = 0.178) nor the funnel plot (Figure 6H) detected publication bias.

**FIGURE 5:**
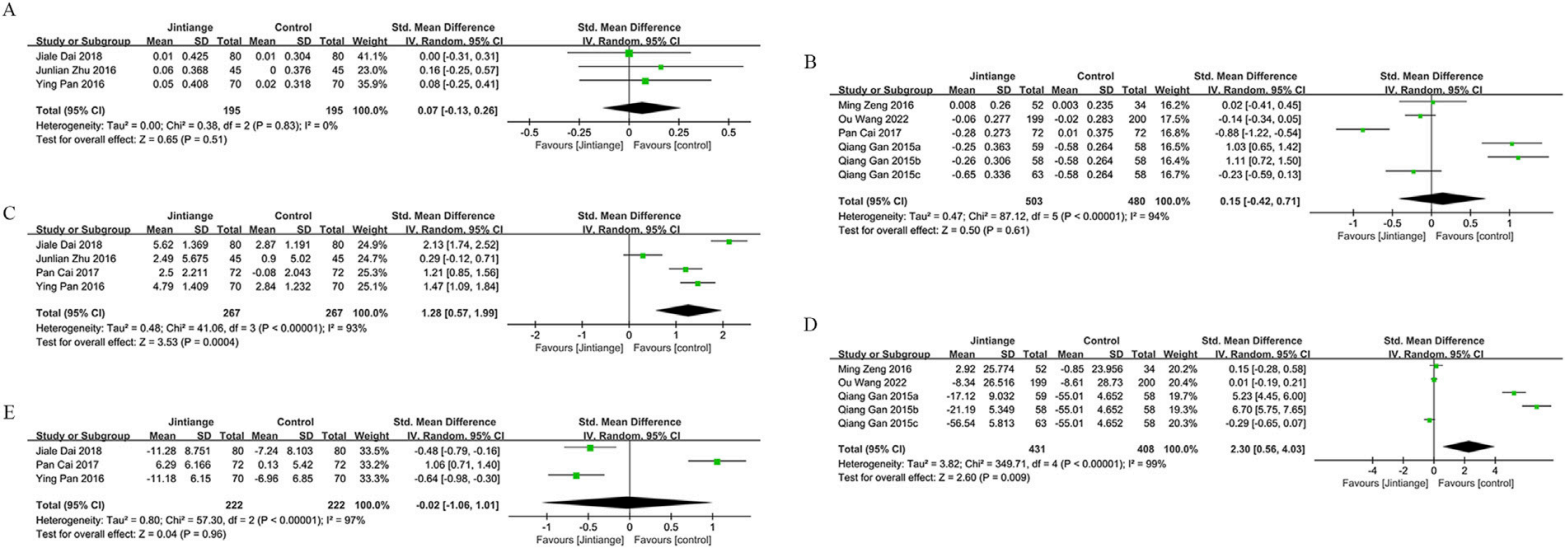
Forest plots illustrate the impact of Jintiange vs the control group on **(A)** Serum Phosphorus, **(B)** CTX, **(C)** BGP, **(D)** PINP, and **(E)** BALP.

**FIGURE 6:**
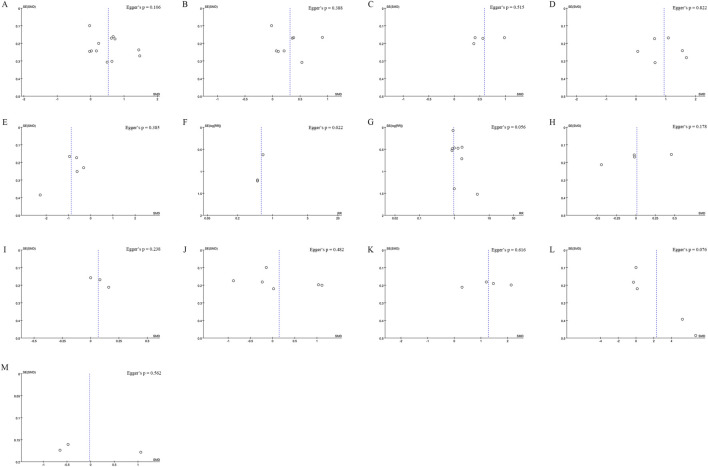
Funnel plot of **(A)** Lumbar Spine BMD **(B)** Femoral Neck BMD **(C)** Greater Trochanter BMD **(D)** Ward’s Triangle BMD **(E)** VAS Score Changes **(F)** Incidence of Fractures **(G)** Incidence of AEs **(H)** Serum Calcium **(I)**Serum Phosphorus **(J)** CTX **(K)** BGP** (L)** PINP **(M)** BALP. Egger’s test p-values are shown in each panel, p < 0.05 indicates significant publication bias.

Three studies reported serum phosphorus data on 390 patients (195 in the Jintiange cohort and 195 in the control cohort). No statistically significant difference was found across the two groups (SMD: 0.07; 95% CI: 0.13, 0.26; p = 0.51), presenting no significant heterogeneity (I^2^ = 0%, p = 0.83) (Figure 5A). Neither Egger’s test (p = 0.238) nor the funnel plot (Figure 6I) showed publication bias.

#### 3.8.2 Bone resorption markers

Data on the serum type I collagen carboxy-terminal peptide (CTX) were available from six studies involving 983 patients (503 in the Jintiange cohort and 480 in the control cohort). The synthesized analysis did not show statistically significant variation in CTX values across groups (SMD: 0.15; 95% CI: 0.42, 0.71; p = 0.61), with significant heterogeneity (I^2^ = 94%, p < 0.00001) (Figure 5B). Neither the funnel plot (Figure 6J) nor Egger’s test (p = 0.482) showed publication bias.

#### 3.8.3 Bone formation markers

Four studies reported bone gla protein (BGP) data on 534 patients (267 in the Jintiange group and 267 in the control group). Our synthesized analysis demonstrated that BGP levels in the Jintiange receivers rose significantly in comparison to baseline, and this rise was markedly higher than that in the control group (SMD: 1.28; 95% CI: 0.57, 1.99; p = 0.0004), showing significant heterogeneity (I^2^ = 93%, p < 0.00001) (Figure 5C). The funnel plot (Figure 6K) and Egger’s test (p = 0.616) did not reveal publication bias.

Data on serum Procollagen type I N-terminal propeptide (PINP) were obtained from five studies on 839 patients (431 in the Jintiange group and 408 in the control group). Our pooled analysis indicated a slight decrease in PINP in the Jintiange cohort than in the control cohort (SMD: 2.30; 95% CI: 0.56, 4.03; p = 0.009), with significant heterogeneity (I^2^ = 99%, p < 0.00001) (Figure 5D). The funnel plot indicated slight publication bias (Figure 6L), but Egger’s test did not display statistically significant publication bias (p = 0.076).

Bone alkaline phosphatase (BALP) data were derived from three studies involving 444 patients (222 in the Jintiange cohort and 222 in the control cohort). Results revealed no statistically significant difference in BALP changes across two groups (SMD = −0.02, 95% CI: 1.06, 1.01, p = 0.96), with significant heterogeneity (I^2^ = 97%, p < 0.00001) (Figure 5E). Neither Egger’s test (p = 0.562) nor the funnel plot (Figure 6M) demonstrated publication bias.

### 3.9 Sensitivity Analysis

**FIGURE 7:**
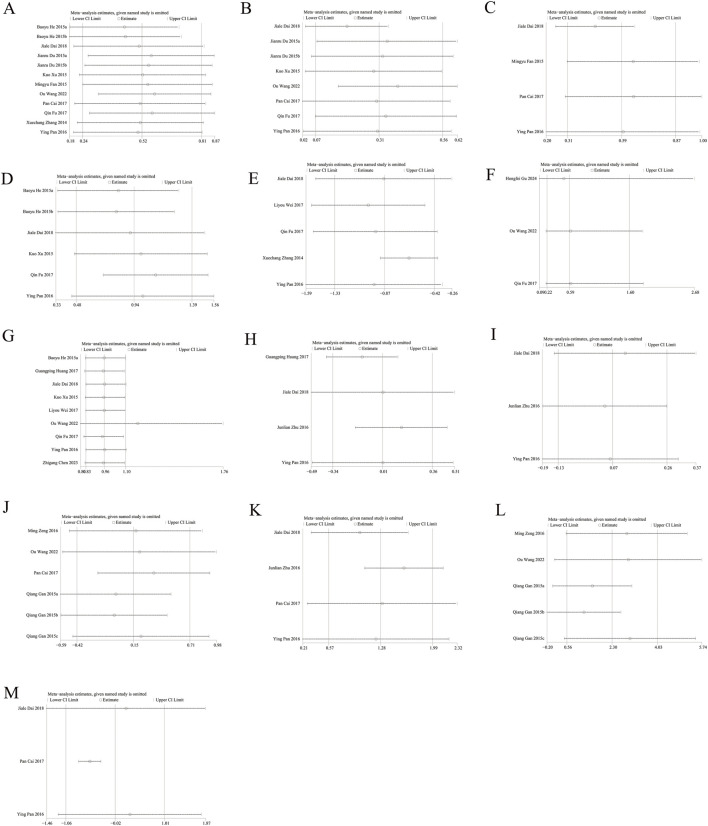
Sensitivity analysis of **(A)** Lumbar spine BMD **(B)** Femoral neck BMD **(C)** Greater trochanter BMD **(D)** Ward’s triangle BMD **(E)** VAS score changes **(F)** incidence of fractures **(G)** Incidence of AEs **(H)** Serum calcium **(I)** Serum phosphorus **(J)** CTX **(K)** BGP **(L)** PINP **(M)** BALP.

Sensitivity analysis performed via Stata showed that the pooled SMD for BMD, VAS scores, serum calcium and phosphorus, CTX, and BGP, as well as the RR for fractures and AEs, were not influenced by any individual study. This suggests that Jintiange indeed improves BMD, alleviates pain, and does not increase the occurrence of AEs with stable and reliable results. Moreover, the removal of the study by Gan et al. (2015) led to changes in the pooled results for PINP, and the exclusion of Pan Cai’s study (2017) affected the pooled results for BALP, suggesting instability in these two bone metabolism markers. Sensitivity analysis results are presented in Figure 7.

### 3.10 Subgroup analysis

To identify the sources of heterogeneity and compare the effects of Jintiange treatment on osteoporosis under different factors, a subgroup analysis was performed on outcomes of interest with sufficient data. First, analysis of lumbar spine BMD data by patient characteristics and intervention duration demonstrated Jintiange’s stronger effect in ameliorating lumbar spine BMD in the old population (SMD: 0.62; 95% CI: 0.33, 0.91; p = 0.0001). Additionally, the effect on lumbar spine BMD improved progressively with longer intervention durations. The most significant improvement seen in interventions lasting more than 6 months (SMD: 0.65; 95% CI: 0.10, 1.19; p = 0.02). However, subgroup analysis by intervention showed no statistically significant difference in lumbar spine BMD with Jintiange monotherapy (SMD: 0.71; 95% CI: 0.12, 1.54; p = 0.1). The heterogeneity in the old subgroup and the Jintiange plus biomedicine subgroup was significantly lowered, which suggests that the high heterogeneity of our outcomes possibly arises from population characteristics or intervention methods. Furthermore, all subgroup results indicated that Jintiange treatment did not increase the occurrence of AEs. Subgroup analysis results are provided in Table 2.

**TABLE 2:** Subgroup analysis of lumbar spine BMD and incidence of AEs.

Subgroup	Change in BMD (lumbar spine)				Adverse events			
	Study	SMD [95%CI]	*P* Value	*I* ^2^	Study	RR [95%CI]	*P* Value	*I* ^2^
Total	12	0.52 [0.24, 0.81]	0.0003	84%	9	0.96 [0.83, 1.10]	0.54	0%
Osteoporosis type								
Senile osteoporosis	2	0.62 [0.33, 0.91]	0.0001	0%	0			
Postmenopausal osteoporosis	3	0.49 [0.08, 0.90]	0.02	72%	4	1.16 [0.72, 1.87]	0.55	0%
Primary osteoporosis (Unclassified)	7	0.53 [0.06, 0.99]	0.00001	89%	5	0.94 [0.81, 1.09]	0.41	0%
Treatment duration								
<6 months	1	0.24 [-0.15, 0.63]	0.23	NA	1	1.29 [0.50, 3.30]	0.6	NA
=6 months	5	0.47 [0.19, 0.75]	0.001	54%	3	0.87 [0.44, 1.73]	0.7	0%
>6 months	6	0.65 [0.10, 1.19]	0.02	91%	5	0.95 [0.83, 1.10]	0.53	0%
Intervention method of Jintiange group								
Combined with conventional treatment	8	0.47 [0.28, 0.66]	0.00001	40%	7	1.23 [0.79, 1.91]	0.36	0%
Monotherapy	4	0.71 [-0.12, 1.54]	0.1	94%	2	0.93 [0.80, 1.08]	0.34	0%

### 3.11 GRADE classification

The GRADE assessment revealed that the quality of evidence for lumbar spine BMD, greater trochanter BMD, Ward’s triangle BMD, VAS scores, AEs, serum phosphorus, and BGP was rated as moderate. In contrast, the quality of evidence for femoral neck BMD, fracture incidence, serum calcium, CTX, PINP, and BALP was rated as low. Detailed GRADE classification results are presented in Table 3.

**TABLE 3:** GRADE rating of each outcome.

No. of studies	Outcomes	SMD/RR	95%CI	*I* ^2^; P value	Risk of bias	Inconsistency	Indirectness	Imprecision	Publication bias	Plausible confounding	Magnitude of effect	Dose-response gradient	GRADE
12	Change in BMD (lumbar spine)	0.52	0.24, 0.81	84%; P < 0.00001	No serious risk	Serious inconsistency	No serious indirectness	No serious imprecision	Undetected	Would not reduce effect	No	No	Moderate
8	Change in BMD (femoral neck)	0.31	0.07, 0.56	72%; P = 0.0006	No serious risk	Serious inconsistency	No serious indirectness	Serious imprecision	Undetected	Would not reduce effect	No	No	Low
4	Change in BMD (greater trochanter of femur)	0.59	0.31, 0.87	61%; P = 0.05	No serious risk	Serious inconsistency	No serious indirectness	No serious imprecision	Undetected	Would not reduce effect	No	No	Moderate
6	Change in BMD (ward’s triangle)	0.94	0.48, 1.39	84%; P < 0.00001	No serious risk	Serious inconsistency	No serious indirectness	No serious imprecision	Undetected	Would not reduce effect	No	No	Moderate
5	Change in VAS score	−0.87	−1.33, −0.42	81%; p = 0.0003	No serious risk	Serious inconsistency	No serious indirectness	No serious imprecision	Undetected	Would not reduce effect	No	No	Moderate
3	Number of fractures	0.59	0.22, 1.60	0%; p = 0.97	No serious risk	No serious inconsistency	No serious indirectness	Serious imprecision	Strongly suspected	Would not reduce effect	No	No	Low
9	Adverse events	0.96	0.83, 1.10	0%; p = 0.86	No serious risk	No serious inconsistency	No serious indirectness	Serious imprecision	Undetected	Would not reduce effect	No	No	Moderate
4	Change in Calcium Concentration	0.23	−0.34, 0.36	76%; p = 0.005	No serious risk	Serious inconsistency	No serious indirectness	Serious imprecision	Undetected	Would not reduce effect	No	No	Low
3	Change in Phosphorus Concentration	0.07	−0.13, 0.26	0%; p = 0.83	No serious risk	No serious inconsistency	No serious indirectness	Serious imprecision	Undetected	Would not reduce effect	No	No	Moderate
6	Change in CTX	0.15	−0.42, 0.71	94%; p < 0.00001	No serious risk	Serious inconsistency	No serious indirectness	Serious imprecision	Undetected	Would not reduce effect	No	No	Low
4	Change in BGP	1.28	0.57,1.99	93%; p < 0.00001	No serious risk	Serious inconsistency	No serious indirectness	No serious imprecision	Undetected	Would not reduce effect	No	No	Moderate
5	Change in PINP	2.30	0.56,4.03	99%; p < 0.00001	No serious risk	Serious inconsistency	No serious indirectness	Serious imprecision	Undetected	Would not reduce effect	No	No	Low
3	Change in BALP	−0.02	−1.06, 1.01	97%, p < 0.00001	No serious risk	Serious inconsistency	No serious indirectness	Serious imprecision	Undetected	Would not reduce effect	No	No	Low

## 4 Discussion

Existing research has demonstrated that TCM, as a remedy derived from nature, has great potential in osteoporosis prevention and treatment thanks to its advantages such as low cost, minimal side effects, and ease of acceptance (Li et al., 2023; Cao et al., 2024). Many clinical studies have reported the remarkable efficacy of Jintiange in treating osteoporosis. However, due to the limited quality and scale of early clinical research, the effectiveness and safety of Jintiange have yet to be robustly confirmed. Internationally, scholars have performed network meta-analyses comparing the efficacy of various CCPP and the combination of Jintiange with other therapies for osteoporosis treatment. Nonetheless, there is scarce direct evidence from RCTs supporting the use of Jintiange in osteoporosis treatment (Sun et al., 2019; Zhao et al., 2021). This meta-analysis is the first evidence-based study based on quality recent RCTs regarding Jintiange for osteoporosis treatment, and it is hoped that it will serve as a valuable reference for future applications and research of this medication.

The pooled analysis demonstrated Jintiange’s significant effect in improving BMD and alleviating pain. BMD is regarded as the gold standard for diagnosing osteoporosis and the primary indicator for assessing treatment efficacy. Under the same conditions, the higher the BMD, the lower the risk of fractures (LeBoff et al., 2022b). Our study showed that the BMD in the Jintiange group was significantly higher in the lumbar spine, femoral neck, greater trochanter, and Ward’s triangle region in comparison to the control group. The results for the lumbar spine and femoral neck aligned with those of Jinlong Zhao et al.’s network meta-analysis (Zhao et al., 2021). Although the pooled results for BMD exhibited considerable heterogeneity, sensitivity analyses failed to identify significant sources of heterogeneity, indicating a degree of stability in the findings. The potential heterogeneity possibly be attributed to differences in study populations (such as sex, age, and comorbidities), variations in pharmacological regimens, and disparities in treatment duration. Furthermore, BMD measurements at sites not included in the pooled analysis due to insufficient outcome data, such as total hip, distal radius, and forearm, also demonstrated more pronounced improvements in the Jingtiange group. However, despite the observed increases in BMD, there was no significant difference in fracture incidence between the two groups. This outcome is subject to publication bias, possibly related to the limited number of included studies and insufficient observation periods for infrequent events like fractures. Therefore, the effect of Jingtiange on fracture risk remains unclear and warrants further investigation. Furthermore, Jintiange had a more advantageous effect in lowering the VAS scores for pain. This result aligns with the conclusions drawn by Sun et al. (2019). Notably, chronic pain in the lower back and joints, often regarded as a “latent symptom”, is more commonly observed in clinical practice than fractures. Such pain can lead to impaired physical function and reduced activity levels, thereby perpetuating a vicious cycle of “reduced activity-bone loss” in affected patients (Logan et al., 2017; Tang et al., 2023).

Regarding safety, the incidence of AEs in the Jintiange group was comparable to that in the control group, with low heterogeneity across studies, indicating that the evidence was stable and reliable. The most frequently observed AEs in the Jintiange cohort included xerostomia (2.6%–12.1%), pyrexia (3.8%–9%), myalgia (1.4%–3%), and constipation (0.8%–2.5%), among others. Although serious AEs were reported by Wang et al. (2022), it was concluded that these events were not related to the studied drug. Moreover, the AEs observed with the combined use of multiple drugs should not be attributed solely to Jintiange. Only the studies by He et al. (2015) and Liang et al. (2022b) included observations where Jintiange was used alone. The AEs reported encompassed dry mouth (7.5%), constipation (0.8%–2.5%), abdominal discomfort (0.8%), elevated gamma-glutamyl transferase (GGT) levels (0.8%), and blepharitis (0.8%). These reactions are more likely to be related to the effects of Jintiange itself, and further clinical trials are necessitated for validation. In summary, compared to conventional biomedicine or its combination with Jintiange, no increase in AEs was noted, and the safety profile was found to be favorable.

In terms of bone metabolism markers, significant differences in serum calcium and phosphate levels were not noted across groups. Serum calcium and phosphate concentrations are regulated by the small intestine, kidneys, and various hormones, and typically remain within normal ranges in patients with primary osteoporosis (Heaney et al., 2010). This finding suggests that Jintiange has no additional impact on calcium and phosphate homeostasis. Bone resorption and formation markers reflect bone turnover in the body and are employed to determine the type of osteoporosis, assess the effects of medications, and predict fracture risk (Schini et al., 2023). These markers exhibit different baseline levels across various sexes, ages, and types of osteoporosis, and possibly vary due to the influence of anti-resorptive or osteoinductive drugs (Eastell and Szulc, 2017). The results of this study show similar changes in CTX levels between the two groups. However, the explanatory power of this finding regarding the effects of Jintiange is limited due to confounding factors such as the use of other anti-resorptive agents and variations in dosing regimens. Similarly, interpreting the results for PINP and BALP presents comparable challenges, particularly due to the instability of these markers observed in the sensitivity analyses. Notably, in the four studies that included BGP, the Jintiange group received a combination treatment, Jintiange administered in addition to the control group regimen, without the involvement of other osteoinductive agents. The findings demonstrated a more pronounced increase in BGP levels with the combined treatment, suggesting that Jintiange exerts an osteogenic effect. However, within this subset of results, all bone metabolism markers except serum phosphorus exhibited considerable heterogeneity, which cannot be overlooked concerning its impact on the stability of the findings.

Subgroup analysis revealed a significant reduction in heterogeneity within the subgroups of senile osteoporosis and Jin Tiange combined with conventional therapy. In the two studies comprising the old subgroup, the Jin Tiange group uniformly employed a combined therapeutic intervention. Therefore, it is postulated that the observed decrease in subgroup heterogeneity possibly be attributed to the minimized variability in intervention modalities. It was also found that treatment durations shorter than 6 months had a weaker effect on lumbar spine BMD, while treatment durations exceeding 6 months showed the best results. This possibly be related to the slow process of bone remodeling and the accumulation of drug effects (Seeman and Martin, 2019). In the subgroup without combined therapy, the improvement in lumbar spine BMD displayed no marked difference in contrast to the control cohort. Based on the foregoing comprehensive analysis, this study suggests that the effect of Jintiange in osteoporosis treatment should not be overestimated. It is recommended to combine Jintiange with conventional therapy and maintain a treatment duration of at least 6 months to improve efficacy without increasing the occurrence of AEs.

The medicinal component of Jintiange, artificial tiger bone powder, is rich in both organic and inorganic substances, including bone collagen, amino acids, calcium, phosphorus, and other trace elements, and can exert anti-osteoporotic effects via regulating the activity of osteoblasts and osteoclasts (Guo et al., 2006). A meta-analysis (LeBoff et al., 2022b) has revealed that, compared to certain other traditional Chinese patent medicines containing herbal components, such as Xianling Gubao Capsule, whose principal ingredients include Epimedium, Dipsacus, and Drynaria, Jintiange demonstrates superior efficacy in Increasing the average BMD at the L2-L4 vertebral levels. Animal experiments have demonstrated that in ovariectomized rats (OVX rats), artificial tiger bone powder promotes osteogenesis and inhibits osteoclastogenesis by modulating signaling pathways like BMP2/SMAD/RUNX2, OPG/RANK/RANKL, and Sirt1/Runx2, increasing collagen content, improving bone microstructure, and enhancing biomechanical strength (Z et al., 2020; Ren et al., 2021). Shen et al. (2022) conducted studies on OVX rats and *in vitro* cells, demonstrating that Jintiange promotes osteoblastic differentiation of BMSCs through BMP and Wnt/β-catenin signaling pathways, and inhibits osteoclastogenesis via downregulation of the NF-κB pathway, thereby preventing bone loss in OVX rats. Fang et al. (2022) employed metabolomics to identify that the anti-osteoporotic effects of Jintiange are linked to the regulation of vitamin B6 and tryptophan metabolism. *In vitro* studies have also proved that Jintiange promotes MC3T3-E1 osteoblast proliferation and inhibits the release of inflammatory cytokines (Li et al., 2022). Liu et al. (2023) removed calcium, phosphorus, and other inorganic elements from artificial tiger bone powder to prepare Jintiange protein. They treated MC3T3-E1 osteoblasts with this protein, finding that it enhances autophagy through PI3K/AKT and ER stress signaling pathways, thereby promoting osteogenesis and inhibiting osteoblast apoptosis. Furthermore, other studies proved that the protective effects of Jintiange on bone tissue also involve mechanisms such as ferroptosis and vascular regeneration (Xie et al., 2024; Yan et al., 2024).

Although the findings of this study support the efficacy and safety of Jintiange in the treatment of osteoporosis, several limitations must be acknowledged. First, the pooled results for the lumbar spine and Ward’s triangle BMD, as well as for biomarkers including CTX, BGP, PINP, and BALP, exhibited substantial heterogeneity. Although sensitivity and subgroup analyses were preliminarily conducted to explore the sources of heterogeneity, the limited number and design of the original studies constrained our ability to fully elucidate potential heterogeneity factors. This necessitates a cautious interpretation of the results, as such heterogeneity possibly affects the robustness of the conclusions. Second, only a minority of the included studies implemented proper allocation concealment and blinding procedures. These methodological limitations possibly have influenced effect size estimates and introduced potential bias. Third, all studies included were conducted exclusively in China, resulting in a homogeneous population sample. Consequently, the generalizability and clinical applicability of the findings to populations in Europe, America, Africa, and other regions remain uncertain. Fourth, the overall intervention duration in the existing studies was insufficient, with follow-up periods not exceeding 12 months. Furthermore, key clinical endpoints such as fracture incidence were inadequately reported, limiting the ability to fully assess the long-term efficacy and safety of the drug. Therefore, future research should focus on conducting more rigorous, multinational, high-quality, multicenter RCTs with larger sample sizes to further validate the results of this study.

## 5 Conclusion

Jintiange improves BMD and alleviates pain in patients with osteoporosis, promotes bone formation by promoting bone Gla protein (BGP), and demonstrates a favorable safety profile. Subgroup analysis suggests that a treatment duration exceeding 6 months is more effective than a shorter duration, and combining Jintiange with conventional treatment is superior to Jintiange monotherapy. In light of certain limitations of the present study, further high-quality research is warranted to strengthen the evidence base and to investigate the long-term efficacy, safety, and impact of Jintiange on fracture incidence.

## Data Availability

The original contributions presented in the study are included in the article/Supplementary Material, further inquiries can be directed to the corresponding author.

## Supplementary material

SUPPLEMENTARY TABLE A1 Search strategy

SUPPLEMENTARY TABLE A2 Detailed report on AEs
