# Supplementary material for: Efficacy and safety of Jintiange in the treatment of osteoporosis: a systematic review and meta-analysis
Source: Front Pharmacol. 2025 Jul 14;16:1592184. doi: 10.3389/fphar.2025.1592184 (PMC12301379; doi:10.3389/fphar.2025.1592184)

Efficacy and safety of Jintiange in the treatment of osteoporosis: a systematic review and meta-analysis

Pubmed-16

((("jintiange" [Supplementary Concept]) OR ("jintiange"[All Fields])) OR ("tiger bone powder"[All Fields])) AND (("Osteoporosis"[Mesh]) OR (((((((((((Osteoporoses) OR (Age-Related Osteoporosis)) OR (Age-Related Osteoporoses)) OR (Age Related Osteoporosis)) OR (Age-Related Bone Loss)) OR (Age-Related Bone Losses)) OR (Senile Osteoporoses)) OR (Senile Osteoporosis)) OR (Involutional Osteoporosis)) OR (Post-Traumatic Osteoporoses)) OR (Post-Traumatic Osteoporosis)))

Embase-27


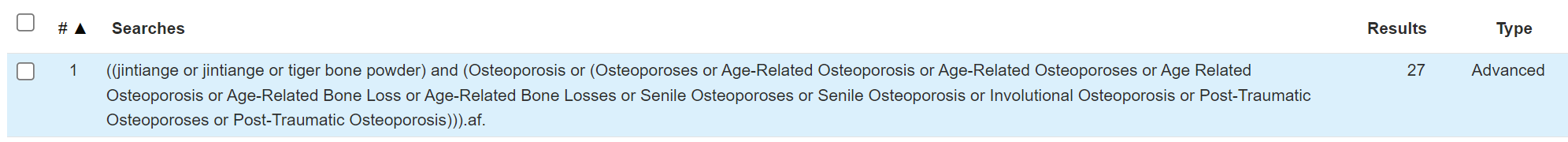


Cochrane-11


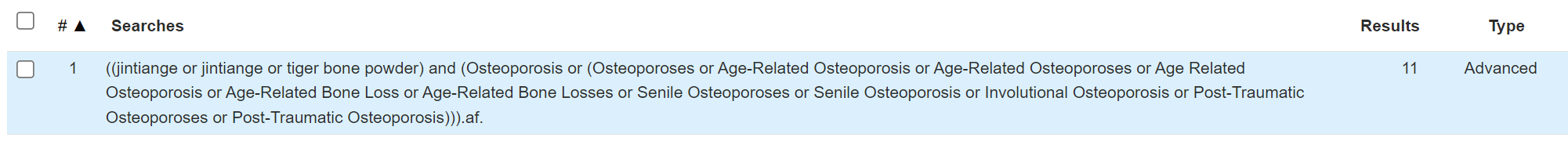


WOS-16

(((jintiange) OR (jintiange)) OR (tiger bone powder)) AND ((Osteoporosis) OR (((((((((((Osteoporoses) OR (Age-Related Osteoporosis)) OR (Age-Related Osteoporoses)) OR (Age Related Osteoporosis)) OR (Age-Related Bone Loss)) OR (Age-Related Bone Losses)) OR (Senile Osteoporoses)) OR (Senile Osteoporosis)) OR (Involutional Osteoporosis)) OR (Post-Traumatic Osteoporoses)) OR (Post-Traumatic Osteoporosis))) (All Fields)

Wanfang-48


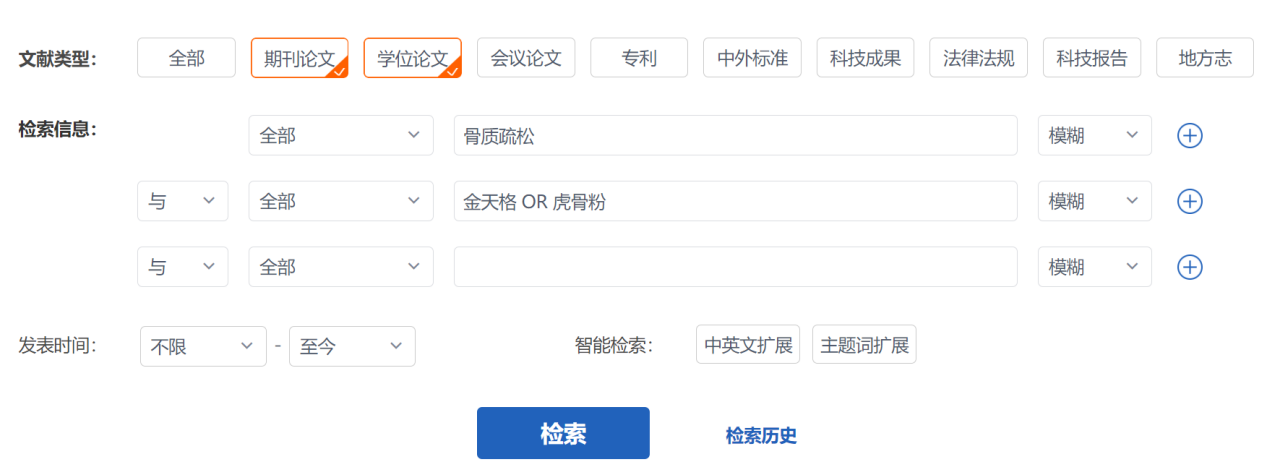


CNKI-230


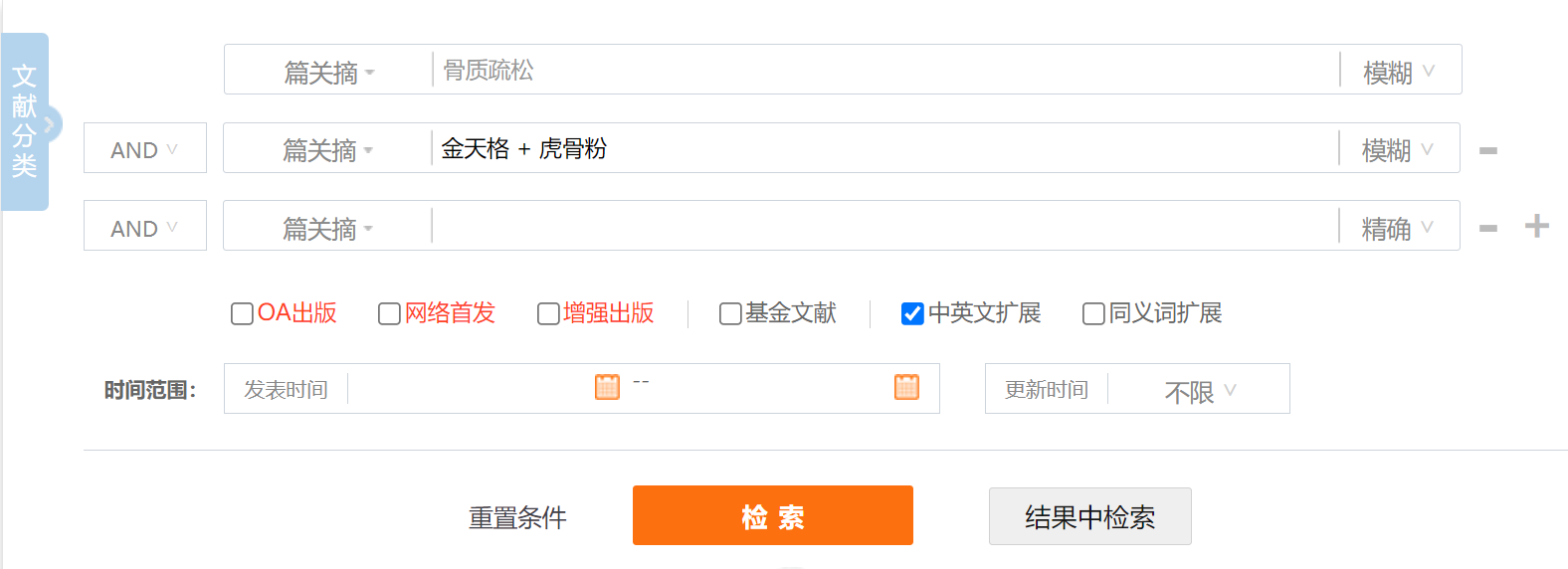

Supplement: Supplementary file 2 [file Table1.docx]
